# Endoscopic observation of acute appendiceal hemorrhage: A case report

**DOI:** 10.1055/a-2512-4208

**Published:** 2025-01-29

**Authors:** Chenyang Jiao, Cuixia Liu, Zhen Yang, Haihua Zhou, Yiwei Fu

**Affiliations:** 1 Department of Gastroenterology, Taizhou Peopleʼs Hospital, Taizhou, China; 2 Department of General Surgery, Taizhou Peopleʼs Hospital, Taizhou, China

A 24-year-old man manifested with bloody stools 10 times within 14 hours without obvious abdominal pain. A computed tomography (CT) scan showed no signs of appendicitis, but the enhanced CT scan revealed contrast agent leakage into the appendiceal lumen ( Fig. 1 **a–c** ). An emergency colonoscopy showed fresh blood at the appendiceal orifice. The diagnosis of appendiceal bleeding was confirmed. However, the underlying cause remained unclear. An ultrathin gastroscope (GIF-XP290N; Olympus, Tokyo, Japan) was then inserted into the appendiceal lumen, revealing the formation of an ulcer with a blood clot at its center ( Video 1 ). The patient underwent a laparoscopic appendectomy ( Fig. 2 **a–d** ). After three months of follow-up, there was no recurrence of rectal bleeding, and hemoglobin levels returned to normal.

**Fig. 1:**
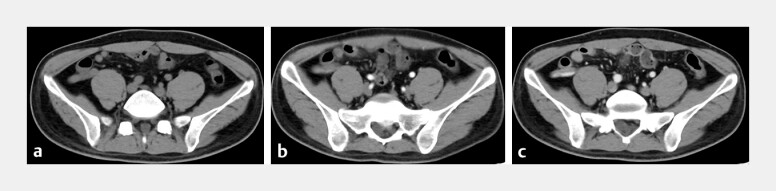
Computed tomography (CT) images of the appendiceal hemorrhage. **a** CT scan without contrast showing no abnormalities. **b** Early arterial phase showing suspicious contrast agent leakage. **c** Late arterial phase showing contrast agent leakage.

**Fig. 2:**
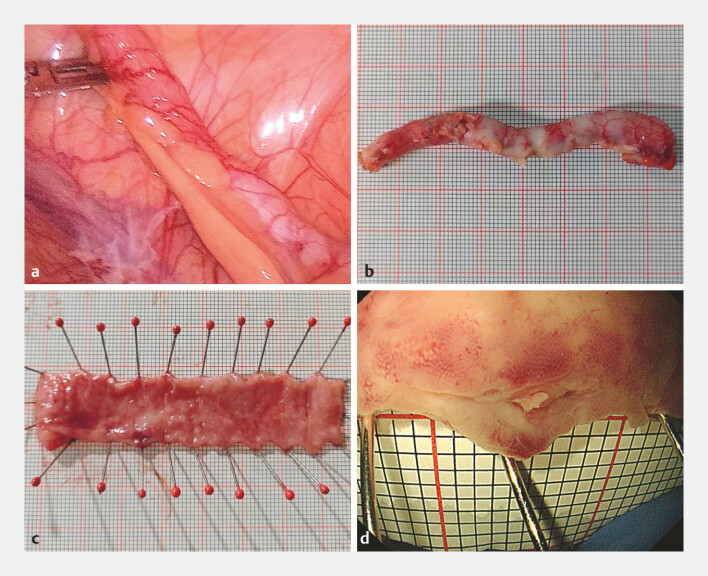
Laparoscopic and postoperative images of the appendix. **a** Laparoscopic exploration shows the appendix with normal size and shape. **b** Serosal surface of the resected appendix specimen. **c** Appendix cut open and flattened, fixed on a specimen board, showing an ulcer. **d** Magnified view of the appendiceal ulcer.

Computed tomography scans and colonoscopy showed active bleeding in the appendix, and the insertion of an ultrathin gastroscope into the appendiceal lumen revealed the formation of ulcers on the appendiceal mucosa.Video 1

To the best of our knowledge, this method has not been reported previously. Current literature reports that appendiceal hemorrhage is caused by various factors leading to the exposure of submucosal vessels due to appendiceal mucosal damage 1 . The primary treatment for appendiceal hemorrhage is an appendectomy, which effectively stops the bleeding 2 . There have been attempts to treat appendiceal hemorrhage by intra-appendiceal stent insertion and detachable snare wrapping 3 . However, this method cannot show the appendiceal mucosa, thus making it impossible to diagnose the cause of the appendiceal hemorrhage. Observing the appendiceal cavity has been challenging due to its narrow lumen, making endoscopic entry difficult. Recently, attempts have been made to use the SpyGlass DS (Boston Scientific, Marlborough, Massachusetts) to observe intra-appendiceal lesions 4 5 . However, the SpyGlass DS requires additional equipment and accessories, making it expensive. In this case, we successfully used an ultrathin gastroscope to observe changes in the appendiceal cavity caused by hemorrhagic lesions. This method has not been reported previously. However, inserting an ultrathin gastroscope into the appendiceal cavity requires a high level of skill from the operator, and since our center has attempted this procedure in only one case, the success rate is currently unknown. Nonetheless, this initial successful attempt that uses an ultrathin endoscope to observe the appendiceal mucosa offers a new approach for diagnosing appendiceal diseases.

Endoscopy_UCTN_Code_CCL_1AD_2AJ

## Notes

Endoscopy E-Videos https://eref.thieme.de/e-videos
*E-Videos* is an open access online section of the journal *Endoscopy* , reporting on interesting cases and new techniques in gastroenterological endoscopy. All papers include a high-quality video and are published with a Creative Commons CC-BY license. Endoscopy E-Videos qualify for HINARI discounts and waivers and eligibility is automatically checked during the submission process. We grant 100% waivers to articles whose corresponding authors are based in Group A countries and 50% waivers to those who are based in Group B countries as classified by Research4Life (see: https://www.research4life.org/access/eligibility/ ). This section has its own submission website at https://mc.manuscriptcentral.com/e-videos .
